# In vivo analysis of hip joint loading on Nordic walking novices

**DOI:** 10.1186/s13018-021-02741-7

**Published:** 2021-10-14

**Authors:** Yannick Palmowski, Srdan Popovic, Simone G. Schuster, Sebastian Hardt, Philipp Damm

**Affiliations:** 1 Center for Musculoskeletal Surgery, Charité – Universitätsmedizin Berlin, Corporate Member of Freie Universität Berlin, Humboldt-Universität Zu Berlin, and Berlin Institute of Health, Chariteplatz 1, 10117 Berlin, Germany; 2 Berlin Institute of Health at Charité – Universitätsmedizin Berlin, Julius Wolff Institute, Augustenburger Platz 1, 13353 Berlin, Germany

## Abstract

**Objective:**

To evaluate the influence of Nordic walking (NW) on hip joint loads in order to determine whether it can be safely performed during postoperative physiotherapy in patients after orthopeadic surgery of the hip.

**Methods:**

Internal hip joint loads were directly measured in vivo in 6 patients using instrumented hip prostheses during NW and ordinary walking (OW). All patients received training in two different NW techniques (double-poling and the diagonal technique) by a certified NW instructor. Measurements were conducted on a treadmill at a speed of 4 km/h on level ground, at 10% inclination and at 10% slope as well as on a level lawn at a self chosen comfortable speed. Resultant contact force (*F*_res_), bending moment (*M*_bend_) and torsional torque (*M*_tors_) were compared between NW and OW as well as between both NW techniques.

**Results:**

Joint loads showed a double peak pattern during all setups. Neither NW technique significantly influenced hip joint loads at the time of the first load peak during contralateral toe-off (CTO), which was also the absolute load peak, in comparison to OW. Compared to OW, double-poling significantly reduced *F*_res_ and *M*_bend_ at the time of the second load peak during the contralateral heel strike (CHS) on level ground both on the treadmill (− 6% and − 7%, respectively) and on the lawn (− 7% and − 9%). At 10% inclination, the diagonal technique increased *F*_res_ and *M*_bend_ at CHS (by + 6% and + 7%), but did not increase the absolute load peak at CTO.

**Conclusion:**

Joint loads during NW are comparable to those of OW. Therefore, NW can be considered a low-impact activity and seems to be safe for patients that are allowed full weight bearing, e.g. during postoperative rehabilitation after THA.

## Introduction

Loading of the musculoskeletal system and joints plays a fundamentdal role in a wide variety of orthopaedic conditions, e.g. regarding osteoarthritis, fractures or after orthopaedic surgery [1]. Mechanical stress of the joints is regarded as an important factor contributing to the development of osteoarthritis, and excessive joint loads may increase the risk of complications such as implant failure, aseptic lossening or implant wear after arthroplasty or internal fixation of fractures [1–7].

Osteoarthritis of the hip joint is among the most common orthopaedic conditions and often results in the necessaity of a total hip arthroplasty (THA) for affected patients [8, 9]. The average age of patients receiving THA surgery has been steadily decreasing during the last years and especially for younger patients, the ability to resume sports is one of the key aims after surgery [10–12]. This has lead to an increase in physical activity of the average patient after joint replacement and thereby also to higher functional demands of the THA [13, 14]. However, there are no evidence based guidelines regarding the return to sports after such surgery, and recommendations regarding the adequate amount and type of physical activity diverge [10, 15]. While excessive sport might lead to accelerated wear, loosening or even fractures, a certain level of physical activity certainly improves quality of life, reduces diverse health risks (e.g. obesity) and may strengthen the muscles supporting the hip joint [16–19]. High impact sports are generally considered to bear the highest risks whereas commonly recommended activities are those that ensure a mobility of the joint, but are not suspected to cause high peak loads, such as cycling aquatic excercice, or Nordic walking (NW) [19–24]. NW has been shown to be effective as cardiovascular training, even though it doesn’t cause a perceived difference in exertion rate compared to ordinary walking [25, 26]. Despite the increase in energy consumption during NW, the ground reaction forces, which were used to calculate the acting joint loads indirectly, have even been reported to be reduced in comparison to ordinary walking as the impact disperses when the poles strike the ground [27]. Such combination of effective cardiovascular training and potentially decreased joint loads would make NW an ideal option for patients with osteoarthritis or after orthopaedic surgery like arthroplasty or fracture fixation.

However, available studies investigating the loads on the musculoskeletal system during NW came to conflicting conclusions as to whether NW increases or decreases joint loads and are all based solely on the measurement of the individual ground reaction forces using force plates or instrumented insoles [28–36]. Using such methods, internal joint loads can only be indirectly estimated through musculoskeletal models [37–40]. A novel approach to directly assess the internal forces is the use of instrumented hip implants, which are able to directly measure in vivo forces and moments in the joint [41–43]. This method has already been used successfully to evaluate other methods of hip joint reduction [21, 44].

The aim of the present study was to clarify the influence of NW on hip joint loads using direct in vivo measurements and to evaluate if NW is an appropriate physical activity for patients after orthopedic surgeries of the hip. For this purpose, we used instrumented hip implants to evaluate in vivo hip joint loads during NW and to compare them to joint loads during ordinary walking. Our hypothesis was that NW does not increase hip joint loads in comparison to ordinary walking and is therefore an ideal option for postoperative physiotherapy after orthopaedic surgeries like THA.

## Material and methods

### Instrumented implants

**Fig. 1:**
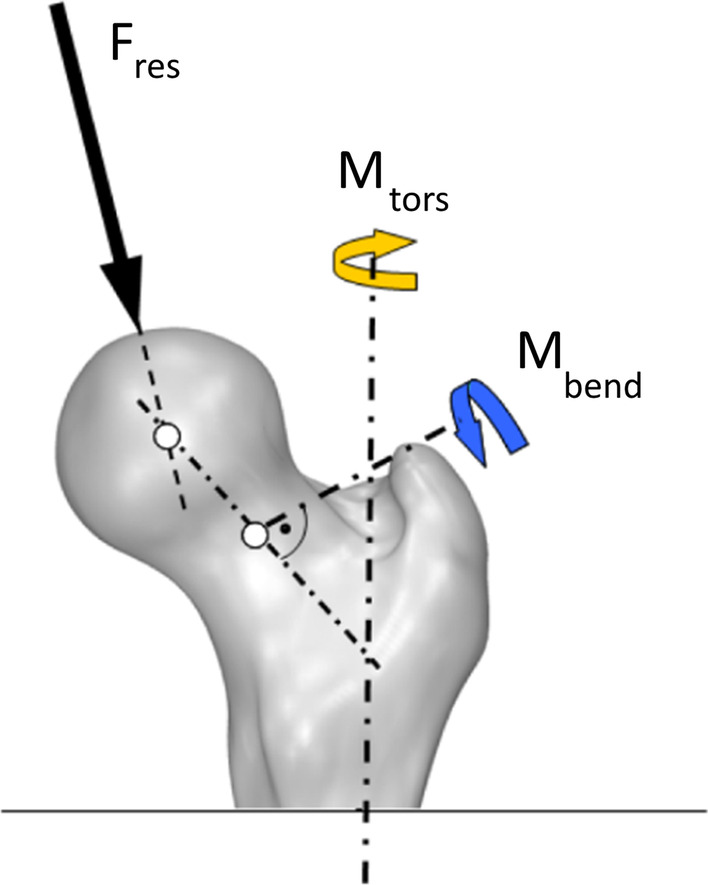
Localisation and direction of the parameters resultant contact force *F*_res_, torsional moment *M*_tors_ and bending moment *M*_bend_

An already described instrumented hip endoprosthesis was used for in vivo measurements of joint loads [43]. It allows to determine the resultant joint force components *F*_*x*_, *F*_*y*_ and *F*_*z*_ as well as the moment components *M*_*x*_, *M*_*y*_ and *M*_*z*_ with a possible measuring error of 1–2% [44]. These force components were subsequently used to calculate the resultant contact force *F*_res_ acting relative to at the femoral head, the bending moment *M*_bend_ acting in the middle of the femur neck, which quantifies most of the stress in the implant neck, as well as the torsion torque *M*_tors_ in the bone-stem-interface respectively in femur shaft axis, which may influence the initial torsional stability of cementless implants in the femur, based on a coordinate system centered at the head of a right side implant (Fig. 1) [45].

### Subjects

Six patients with an instrumented hip prosthesis were willing to participate and are included in this study (Table 1). All patients underwent hip replacement surgery using using the direct lateral approach These patients are part of a worldwide unique collective of 10 patients with instrumented implants (all of which were asked to participate in the present study) and have already participated in other previously published studies [21, 24, 45–52]. The measurements were performed between 8 and 32 months after surgery. In all patients, the indication for the THA had been osteoarthritis and all of them were NW novices.

**Table 1:** Subjects participating

Participant	Gender	Age* (years)	Weight* (kg)	Height* (cm)	BMI* (kg/m^2^)	Time since surgery* (months)	Pole length (cm)
H2R	M	64	80	172	27	32	118
H5L	W	64	87	168	30.8	24	118
H6R	M	69	86	176	27.8	17	120
H7R	M	54	92	179	28.7	19	123
H8L	M	56	86	178	27.1	14	123
H9L	M	54	118	181	36.3	8	120
MW ± STD		60 ± 6	91 ± 14	176 ± 5	30 ± 3	19 ± 8	120 ± 2

^*^on the day of the measurements

### In vivo load measurements

For all in vivo load measurements, the same NW Poles (Flash Vario®, model 2012, Leki, Kirchheim, Germany) were used. The standard length of the poles was 120 cm, but could be individually adapted by ± 5 cm to fit the patients’ proportions (Table 1).

**Fig. 2:**
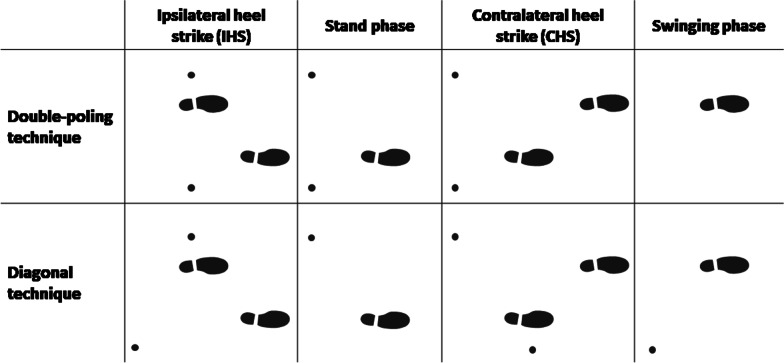
Nordic Walking techniques (referring to the right leg)

The in vivo load data were collected during walking and NW on a level lawn at self-chosen speed as well as during treadmill walking on level ground with 4 km/h. Furthermore, the in vivo loads were determined at ordinary walking respectively NW with 10% inclination and with 10% slope at 4 km/h. For each measurement two different NW techniques, the diagonal technique and double-poling, were performed (Fig. 2) and the resultant in vivo joint loads were examined in comparison to ordinary walking without poles.

For the diagonal technique, each arm comes forward together with the contralateral leg and the pole is put onto the ground around the same time as the contralateral heel. For the double-poling technique, both poles come forward together with the same leg and are put onto the ground at the same time, either in a 2:1 or a 3:1 rhythm. In our study, a 2:1 rhythm was performed in which the NW poles always touched the ground at the same time as the heel of the ipsilateral leg.

As none of the participants had prior experience with NW, each received an individual instruction by a licensed NW instructor before the measurements. Additionally, all measurements were performed under the guidance of the same instructor as well. Selected trials of each measurement are published and can be downloaded at the public data base www.OrthoLoad.com. After familiarization, around 30 steps were measured for each setup and patient, which were averaged first individually and then for the whole cohort.

### Data collection and evaluation

During the in vivo load measurements, all patients were filmed continually and simultaneously the in vivo data from the instrumented prostheses were stored on the same ditigital video tape. Details regarding the methods and the external measurement system have already been described previously [53].

The in vivo determined time-load patters of each patient were averaged intra-individually and separately for each walking technique using a time warping method [54]. Subsequently, an inter-individual average was calculated for each load component based on the individual averages to determine time load patterns of an average subject for each walking techniques. Peak loads at characteristic time points were determined for each load component. All in vivo measured forces and moments were given as percent of the bodyweight %BW respectively %BWm.

Statistical analysis was performed using IBM SPSS Statistics 20.0/21.0 (New York, USA). Non-parametric Wilcoxon Test was used to compare the mean values with respect to the different setups. The significance level was set to *p* ≤ 0.05.

## Results

### Walking on a level lawn

In the first part of the in vivo load measurement the participants walked on a level lawn with and without NW poles at a self-chosen comfortable speed (Table 2). On average, the patients walked faster when using NW poles than when walking without. The difference in walking speed between NW and ordinary walking varied inter-individually between + 1.1 and − 0.26 km/h.

**Table 2:** Walking speed during ordinary walking and Nordic walking (NW) on a lawn with two different techniques

	Ordinary walking (km/h)	NW-diagonal (km/h)	NW-double-poling (km/h)
H2R	4.06	4.54	3.80
H5L	4.73	4.60	4.81
H6R	3.71	4.81	4.79
H7R	4.62	4.75	4.47
H8L	3.97	4.00	4.35
H9L	5.04	5.25	5.55
Mean	4.35	4.66	4.63

**Fig. 3:**
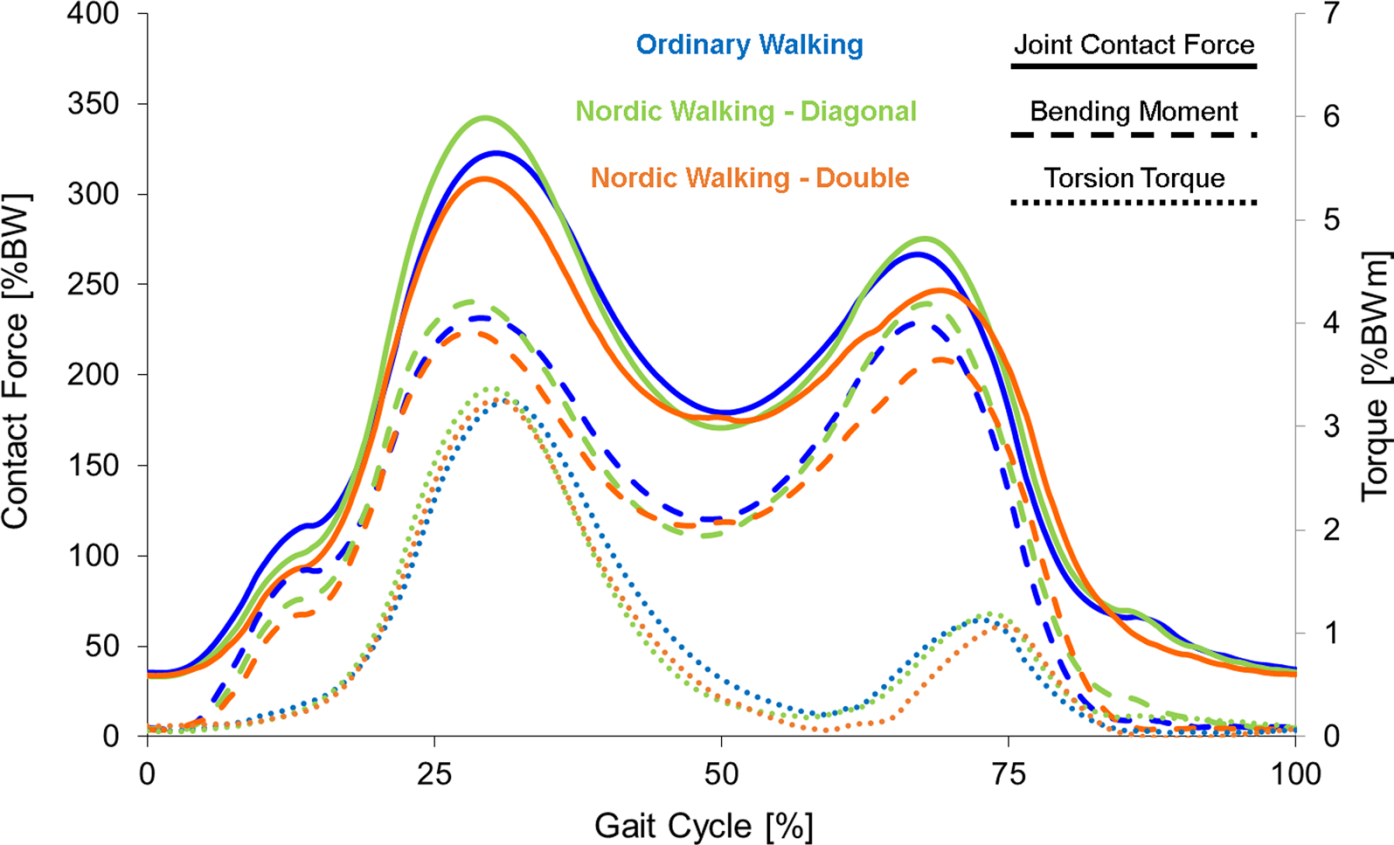
Hip joint loads during walking on level lawn

The in vivo measured joint loads *F*_res_ and *M*_bend_ show a double-peaked pattern that is characteristic for walking (Fig. 3). The first maximum occurs at the time of contralateral toe-off (CTO) and is followed by a second maximum at the time of contralateral heel strike (CHS). *M*_tors_ shows a clear maximum corresponding to the first maxima of *F*_res_ and *M*_bend_ at CTO. Overall the curves show similar patterns for ordinary walking and the two NW techniques.

Absolute averaged peak loads for each parameter occurred at CTO with 323%BW for *F*_res_, 4.06%BWm for *M*_bend_ and 3.3%BWm for *M*_tors_, whereas joint loads at CHS were lower with 267%BW for *F*_res_ and 4.01%BWm for *M*_bend_ (values for ordinary walking). For all three examined parameters the overall highest joint loads were observed during NW using the diagonal technique at the time of contralateral toe off (CTO), with on average 343%BW for *F*_res_, 4.22%BWm for *M*_bend_ and 3.44%BWm for *M*_tors_. NW with either technique did not have a significant influence on absolute peak joint loads at CTO compared to ordinary walking. Double-poling NW technique resulted in the overall lowest joint loads with 309%BW (CTO) and 248%BW (CHS) for *F*_res_, 3.92%BWm (CTO) and 3.66%BWm (CHS) for *M*_bend_, and 3.32%BWm for *M*_tors_. Double-poling significantly reduced the second (lower) maxima for both *F*_res_ and *M*_bend_ by − 7% and − 9% compared to ordinary walking (Table 3). The respective first (higher) maxima showed a trend towards a load reduction for double-poling, while *M*_tors_ does not change relevantly.

**Table 3:** Averaged in vivo measured hip joint loads during ordinary walking and Nordic Walking

	*F*_res_						*M*_bend_						*M*_tors_		
	1. Max (%BW)	*Δ* (%)	*p*	2. Max (%BW)	*Δ* (%)	*p*	1. Max (%BWm)	*Δ* (%)	*p*	2. Max (%BWm)	*Δ* (%)	*p*	Abs_max (%BWm)	*Δ* (%)	*p*
*Ordinary walking*															
Level lawn	323 ± 45			267 ± 39			4.06 ± 00.8			4.01 ± 0.6			3.30 ± 0.4		
Level ground (treadmill)	300 ± 39			263 ± 35			3.89 ± 0.8			3.73 ± 0.6			2.54 ± 0.4		
10% Inclination	319 ± 40			242 ± 38			3.99 ± 0.8			3.41 ± 0.5			3.32 ± 0.5		
10% Slope	335 ± 41			253 ± 39			4.29 ± 0.7			3.58 ± 0.6			3.38 ± 0.6		
*Diagonal technique*															
Level lawn	343 ± 52	+ 6	0.173	276 ± 35	+ 3	0.058	4.22 ± 0.9	+ 4	0.463	4.19 ± 0.5	+ 5	0.116	3.44 ± 0.6	+ 4	0.463
Level ground (treadmill)	308 ± 48	+ 3	0.115	267 ± 37	+ 1	0.463	3.91 ± 1	+ 1	0.753	3.94 ± 0.6	+ 6	0.116	2.62 ± 0.5	+ 3	0.249
10% Inclination	328 ± 43	+ 3	0.463	256 ± 35	+ 6	0.028	4.03 ± 0.76	+ 1	0.917	3.64 ± 0.6	+ 7	0.026	3.12 ± 0.7	− 6	0.173
10% Slope	340 ± 45	+ 2	0.600	259 ± 33	+ 2	0.141	4.29 ± 0.68	0	0.753	3.63 ± 0.6	+ 1	0.463	3.46 ± 0.5	+ 2	0.600
*Double-poling*															
Level lawn	309 ± 50	− 4	0.046	248 ± 37	− 7	0.028	3.92 ± 0.9	− 3	0.344	3.66 ± 0.5	− 9	0.028	3.32 ± 0.6	+ 1	0.753
Level ground (treadmill)	297 ± 36	− 1	0.674	247 ± 28	− 6	0.043	3.83 ± 0.9	− 1	0.463	3.48 ± 0.5	− 7	0.046	2.69 ± 0.5	+ 6	0.400
10% Inclination	313 ± 51	− 2	0.600	249 ± 46	+ 3	0.600	3.83 ± 0.86	− 4	0.075	3.32 ± 0.6	− 3	0.249	3.13 ± 0.8	− 5	0.463
10% Slope	325 ± 41	− 3	0.141	242 ± 39	− 4	0.249	4.15 ± 0.64	− 3	0.345	3.38 ± 0.6	− 6	0.116	3.44 ± 0.6	+ 2	0.832

*F*_res_: resultant contact force; *M*_bend_: bending moment; *M*_tors_: torsional moment; *Δ*: relative difference compared to ordinary walking at the same situation (level, inclination and slope); *p*: *p* value for the comparison of joint loads with ordinary walking

### Treadmill walking on level ground at 4 km/h

For treadmill walking on level ground at 4 km/h, *F*_res_ and *M*_bend_ also show the double-peaked pattern that is characteristic for walking. The absolute peak joint loads during ordinary walking on treadmill with 4 km/h were on average 300%BW for *F*_res_, 3.89%BWm for *M*_bend_ and 2.54%BWm for *M*_tors_ (Table 3). This equals a reduction of joint forces of − 7.1% for *F*_res_, − 4.2% for *M*_bend_ and − 23% for *M*_tors_ compared to walking on a lawn.

Overall the curves show a similar pattern for ordinary walking and the two NW techniques. Neither of the two NW techniques significantly influenced the absolute peak loads at CTO. NW using the diagonal technique shows a trend towards increased joint loads, which is most pronounced for *M*_bend_ at CHS with + 6%, but does not reach significance level (Table 3). Double-poling significantly reduces the second (lower) peak at CHS of *F*_res_ by − 6% and *M*_bend_ by − 7%, but shows a trend towards an increase in *M*_tors_ of + 6%.

### Walking with inclination and slope on a treadmill at 4 km/h

Walking with inclination and slope showed the same double-peaked pattern for *F*_res_ and *M*_bend_ as walking on level ground, both for ordinary walking and for NW. However, the first maximum (at CTO) increased for both parameters by up to + 11.7% (ordinary walking, *F*_res_) and + 10.3% (ordinary walking, *M*_bend_) at a 10% slope compared to walking on a level treadmill. For all three examined parameters (*F*_res_, *M*_bend_ and *M*_tors_), the highest joint loads were observed at the time of CTO using the diagonal technique at 10% slope with 340%BW for *F*_res_, 4.29%BWm for *M*_bend_ and 3.46%BWm for *M*_tors_. NW with either technique did not significantly change absolute peak joint loads at CTO compared to ordinary walking. Using the diagonal technique at 10% inclination significantly increased the second maxima (at CHS) of both *F*_res_ by + 6% and *M*_bend_ by + 7%, compared to ordinary walking with 10% inclination. For a slope of 10%, the in vivo hip joint loads for the diagonal technique did not significantly differ from ordinary walking with the same slope. Double-poling did not significantly change any of the examined joint loads at 10% inclination or at 10% slope in comparison to ordinary walking.

### Comparison of joint loads between diagonal technique and double-poling (all setups)

When comparing in vivo joint loads between both NW techniques, diagonal versus double poling, *F*_res_ and *M*_bend_ show a general trend towards higher values for the diagonal technique (Table 4). The highest differences were observed during walking on a level lawn, where double-poling significantly reduced the absoluted peak of *F*_res_ at CTO compared to the diagonal technique. Additionally, it significantly reduced *F*_res_ at CHS by − 10% and *M*_bend_ at CHS by − 13% on a level lawn. There were no significant differences between both techniques regarding *M*_tors_.

**Table 4:** Percentual differences in hip joint loads between diagonal technique and double-poling

	*F*_res_				*M*_bend_				*M*_tors_	
	1. Max		2. Max		1. Max		2. Max		Absolute maximum	
	*Δ* (%)	*p*	*Δ* (%)	*p*	*Δ* (%)	*p*	*Δ* (%)	*p*	*Δ* (%)	*p*
Level ground	− 4	0.141	− 7	0.028	− 2	0.345	− 12	0.028	− 3	0.600
10% Inclination	− 5	0.249	− 3	0.345	− 5	0.046	− 9	0.028	0	0.600
10% Slope	− 4	0.116	− 7	0.075	− 3	0.172	− 7	0.046	− 1	0.345
Lawn	− 10	0.028	− 10	0.028	− 7	0.116	− 13	0.028	− 3	0.753

Differences are presented in relation to the values of the diagonal technique. *F*_res_: resultant contact force; *M*_bend_: bending moment; *M*_tors_: torsional moment

## Discussion

In this study we examined hip joint loads in vivo during ordinary walking and NW at different inclinations and using two different NW techniques. The aim was to clarify the influence of NW on hip joint loads and to determine in vivo whether NW is an appropriate physical activity during postoperative rehabilitation in patients after orthopaedic surgery of the hip. For this purpose, we used instrumented implants to conduct direct measurements of the respective joint loads during NW in vivo. It should be noted that this is an important difference to most existing studies we compare our data to, as the majority of them calculate internal forces from external measurement using the musculoskeletal models [28–36]. Such mathematical models may come to different results since they rely on the measurement of external instead of intrinsicly generated forces.

Regarding the influence of NW on in vivo joint loads during level treadmill walking, neither of the two NW techniques lead to a significant increase in joint loads. The double poling technique resulted in a slight but significant decrease of joint contact Force *F*_res_ (− 6%) as well as the bending moment *M*_bend_ on the femur neck (− 7%) at the time of the contralateral heel strike respectively the second (lower) peak of the typical load pattern, while absolute peak loads at CTO were not significantly influenced. Similar results were observed for walking on a level lawn at self chosen velocity, where double poling also slightly, yet significantly reduced the second (lower) peak of both *F*_res_ and *M*_bend_ by − 7% and − 9%, respectively. The diagonal technique on the other hand showed a trend towards higher joint loads of + 3% for the absolute peak loads of *F*_res_ and *M*_tors_, which however did not reach significance. It should be noted that the average walking velocity was 0.31 km/h (+ 7.1%) faster than for normal walking. This might explain the trend towards higher joint loads, as an increase of up to + 6% should be expected merely due to the higher walking speed [55]. From these results we conclude that NW on a level lawn using the diagonal technique does not relevantly increase hip joint loads. These results are in line with those from two previous studies, that reported either a slight reduction of joint forces or no difference in joint loads for NW on level ground [36, 56]. One study with 15 patients reported siginificantly higher lower extremity joint loads for NW compared to ordinary walking [57]. However, this study focused on the knee instead of the hip and was performed at a much higher walking speed than our study (7.2 km/h versus 4.4–4.7 km/h) [57].

Similar to walking on level ground, NW with either technique at 10% slope or 10% inclination did not significantly influence hip joint loads in vivo compared to ordinary walking under the same conditions. Our results showed an increase of the second (lower) peak (CHS) for *F*_res_ and *M*_bend_ at 10% inclination for the diagonal technique. However, the increases were overall moderate (≤ + 7%) and hardly affected the absolute peak loads, which occurred at the first maximum for both *F*_res_ and *M*_bend_ and only showed a trend towards an increase of ≤  + 3%. Since the peak loads were hardly influenced by NW, an elevated risk due to NW using the diagonal rechnique after orthopaedic surgery seems rather unlikely. To the best of our knowledge, this is the first study to examine the influence of NW on hip joint forces at inclined surfaces in vivo, so that a comparison to existing studies is difficult. The influence of NW on lower extremity joint forces during walking downhill has been examined by one other study so far, which used 3:1 double-poling at a slope of 25% [58]. This study reported a reduction of ground reaction forces and calculated knee joint loads of 12–25% for NW. Although we did not observe a significant reduction of joint forces for NW at 10% slope, our results confirm that NW does not lead to increased joint loads at 10% slope and can therefore safely be performed after THA. When comparing the two techniques, double-poling showed overall lower joint loads for both *F*_res_ and *M*_bend_, ranging up to a difference of − 13% for *M*_bend_ at CHS and − 10% for *F*_res_ at CTO and CHS on a level lawn. *M*_tors_ did not significantly differ between the two techniques. These results suggest that in situations where the avoidance of elevated loads is particularly important, e.g. in the early postoperative phase after orthopaedic surgery, double-poling may be preferred over the diagonal technique. However, it needs to be kept in mind that we only examined forces of the ipsilateral leg, which is put onto the ground at the same time as the poles. No conclusion can be drawn regarding the influence of double-poling on the contralateral leg.

Overall, our results show that NW on level ground does not lead to a relevant increase in hip joint forces. Therefore, we suggest that NW can be safely performed on level ground or at moderate inclinations and slopes by any patient who is allowed ordinary walking with the same walking velocity, e.g. during postoperative rehabilitation after THA. The exact time at which full weight bearing is allowed depends on the circumstances of the surgery (e.g. primary THA vs. revision THA) and is usually decided by the surgeon. For routine primary THA, early full weight bearing has been reported to be safe and is becoming increasingly popular, so that NW could already be introduced in early postoperative stages as soon as patients are able to walk comfortably without crutches [59]. This is of particular interest as NW has been shown to be a good stimulus for the circulation through the use of upper body musculature [60]. Despite the resulting increase in energy expenditure, NW has been reported to cause no perceived difference in exertion rate, which may offer a very convenient way to start sports again after surgery or injury [26]. This is supported by the observations in our study as well as in published literature, where the patients’ self-chosen comfortable walking speed was faster for NW than for ordinary walking [27]. One study even reported NW to be superior to both strength training and home-based exercises for improving function in patients with hip osteoarthritis [61]. Therefore, NW also seems like an ideal option for younger patients that desire a fast return to physical activity after THA. Furthermore, NW has been shown to improve gait asymmetry [62]. Gait asymmetry occurs in patients with lower extremity osteoarthritis, often persists after THA and is suspected to be associated with accelerated implant wear, making its correction an important aim [63–65]. Even though NW seems generally safe, it should be noted that it might still lead to an elevated risk through postoperative disturbances of balance or the increase in walking speed that we observed compared to ordinary walking. A higher walking speed directly increases hip joint loads and might also increase the risk of falls [55]. Therefore, it seems advisable to only recommend NW once patients are able to comfortably walk without crutches and to remind them to carefully consider their walking speed.

Sports that are typically recommended for early postoperative physiotherapy include low-impact activities such as cycling and aquatic exercises [10, 15]. For dynamic aquatic exercises, a previous in vivo study using instrumented implants reported resultant joint forces in the hip joint of 280%BW [21]. These values are similar to those we observed for double-poling on a treadmill with level ground, where *F*_res_ was 297%BW. However, joint loads during aquatic exercises could be lowered considerably if only non-weight-bearing or weight-bearing activities were performed, with values of 106–155%BW. The effect of cycling on hip joint loads has been examined by another study, which reported resultant hip joint forces of 118%BW for an ergometer at 110 W [24]. Consequently, cycling on an ergometer as well aquatic exercises using non-weight-bearing and weight-bearing activities might be more suitable for patients with load reductions, whereas NW and dynamic aquatic exercises seem to be safe as soon as full weight-bearing is allowed.

Despite our efforts for a rigorous methodology, there are some limitations to our study. The patient cohort is rather small with six participants, five of them male. Larger studies with a more equal representation of male and female patients would be desirable to confirm our results, but are be difficult to realize due to the highly complex methodology. For the measurements on a level lawn, walking velocities were not standardized and each patient chose an individual comfortable walking velocity instead. However, the individual walking velocities only differed by up to − 0.8 or + 0.9 km/h from the respective average. Yet, we would like to underline that the walking speed only differed between the participants during walking on the lawn, while all other measurements were performed at the same fixed walking speed of 4 km/h for all participants. Treadmill walking at a defined walking velocity on the other hand may have been unfamiliar for some of the participants. All patients in this study were NW novices. This ensures a good applicability to patients looking for allowed activies after orthopedic surgeries, as the majority of these may not have previous NW experience. However, it is possible that joint loads would differ in a more experienced cohort. We suspect joint loads to rather decrease than increase in such patients, as more experience will likely lead to a more efficient technique. Furthermore, due to the use of instrumented implants for the in vivo load measurements, only patients with previous THA could be included. Even though it seems likely that the effects of NW on joint loads are similar in healthy patient, no definite conclusions can be drawn from our study.

## Conclusion

The study presents for the first time in vivo hip joint loads during NW with two different techniques. These unique in vivo data suggest that NW on level ground and at moderate inclinations or slopes does not relevantly change hip joint loads compared to normal walking at the same conditions. Thus, NW at normal walking speed can be considered a low-impact sport and seems to be a safe postoperative activity for patients that are allowed full weight bearing. However, it should be kept in mind that NW is often performed at a higher walking speed than ordinary walking, which may cause increased joint loads.

## Data Availability

Selected trials of each measurement are published and can be downloaded at the public data base www.OrthoLoad.com.
